# Dibutyltin(IV) and Tributyltin(IV) Derivatives of *meso*-Tetra(4-sulfonatophenyl)porphine Inhibit the Growth and the Migration of Human Melanoma Cells

**DOI:** 10.3390/cells8121547

**Published:** 2019-11-30

**Authors:** Francesca Costantini, Fabiana Di Leo, Caterina Di Sano, Tiziana Fiore, Claudia Pellerito, Giovanna Barbieri

**Affiliations:** 1 Istituto per la Ricerca e l’Innovazione Biomedica (IRIB), Consiglio Nazionale delle Ricerche (CNR), 90146 Palermo, Italy; francesca.costantini@irib.cnr.it (F.C.); fabyfdl@gmail.com (F.D.L.); caterina.disano@irib.cnr.it (C.D.S.); 2 Dipartimento di Fisica e Chimica, Università degli Studi di Palermo, 90128 Palermo, Italy; tiziana.fiore@unipa.it (T.F.); claudia.pellerito@unipa.it (C.P.); 3 Consorzio Interuniversitario di Ricerca in Chimica dei Metalli nei Sistemi Biologici (C.I.R.C.M.S.B.), 1-70121 Bari, Italy

**Keywords:** melanoma, organotin(IV), cellular growth, BRAF, cell cycle, migration

## Abstract

Melanoma is the most aggressive and deadly form of skin cancer, which is largely due to its propensity to metastasize. Therefore, with the aim to inhibit the growth and the metastatic dissemination of melanoma cells and to provide a novel treatment option, we studied the effects of the melanoma treatment with two organotin(IV) complexes of the *meso*-tetra(4-sulfonato-phenyl)porphine, namely (Bu_2_Sn)_2_TPPS and (Bu_3_Sn)_4_TPPS. In particular, we showed that nanomolar concentrations of (Bu_2_Sn)_2_TPPS and (Bu_3_Sn)_4_TPPS are sufficient to inhibit melanoma cell growth, to increase the expression of the full-length poly (ADP-ribose) polymerase (PARP-1), to induce the cell cycle arrest respectively at G2/M and G0/G1 through the inhibition of the Cyclin D1 expression and to inhibit cell colony formation. Nanomolar concentrations of (Bu_2_Sn)_2_TPPS and (Bu_3_Sn)_4_TPPS are also sufficient to inhibit the melanoma cell migration and the expression of some adhesion receptors. Moreover, we report that (Bu_2_Sn)_2_TPPS and (Bu_3_Sn)_4_TPPS act downstream of BRAF, mainly bypassing its functions, but targeting the STAT3 signalling protein. Finally, these results suggest that (Bu_2_Sn)_2_TPPS and (Bu_3_Sn)_4_TPPS may be effective therapeutic strategies for their role in the inhibition of melanoma growth and migration.

## 1. Introduction

**Figure 1:**
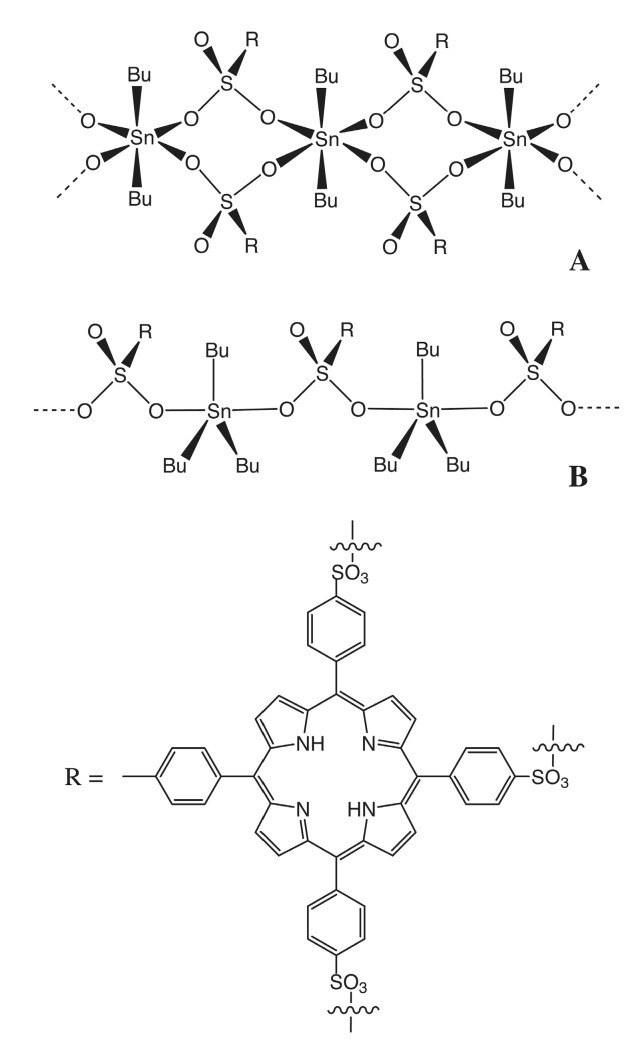
Polymeric octahedral configuration for (Bu_2_Sn)_2_TPPS (**A**) and polymeric trigonal-bipyramidal configuration for (Bu_3_Sn)_4_TPPS (**B**), as characterized in the solid state [8].

Melanoma is the most aggressive form of skin cancer and unfortunately one of the most lethal cancers worldwide, whose incidence rates are increasing rapidly in western populations [1,2]. Indeed, the acquisition of invasive behaviour by metastatic melanoma cells is the key transition involved in the decrease of melanoma patient survival rates. In particular, the metastatic transformation of melanoma cells is a stepwise mechanism that depends on the increase of survival molecule mutations that inhibit apoptotic pathways, allowing survival to hypoxia and oxidative stress [3] and increasing the cellular migration and dissemination, which leads to the transformation of melanocytes into metastatic melanoma. Indeed, despite the fact targeted therapies and immunotherapies have changed significantly the metastatic melanoma treatment landscape [4], significant morbidity and mortality are still associated with metastatic disease, and in fact only 32% of patients with metastatic melanoma have a 5-year median survival rate, compared to almost 93% of patients with early melanoma lesions [5]. Therefore, the melanoma field is in great need of studies to identify new efficient therapies suitable for overcoming the intrinsic survival features acquired by melanoma cells to promote proliferation and invasion. In this contest a group of organometallic compounds, the organotin complexes, widely accepted in oncology as potential anticancer drugs, is very interesting [6,7]. The general formula of organotin complexes is R_n_SnX_(4−n)_, with n = 1 to 4, where the tin atom is covalently bound to one or more organic alkyl or aryl groups (R) as well as to inorganic or organic ligands (X) [6]. The nature and the number of the alkyl or aryl groups bound to the tin atoms of the organotin(IV) are crucial for the anticancer activity of the complexes, while the ligands play a key role addressing the organotin complexes to the target sites of the cells and modulating its cytotoxic activity [8,9,10]. Indeed, tin-based compounds can bind some membrane and cytoplasmic proteins such as receptors and glycoproteins, can cross lipid bilayer membranes and reach the nucleus, where they can directly interact with the DNA bases through intercalation, can also interact with the functional groups of the DNA grooves and with the phosphate of the DNA phosphodiester backbones as anchoring sites for the tin [11,12,13]. The consequences of these interactions are both the structural and functional alterations of the membranes and the induction of DNA damage that leads to the blockage of cell division and cell growth of organotin(IV)-treated cancer cells [6,8,9,10,14,15,16]. Moreover, the ligand molecules of the organotin(IV) complexes, modifying the reactivity, the lipophilicity, the configuration and the molecular structure of the complexes and therefore their binding activity and functions, play a significant role in modulating the anti-tumour activity of the organotin(IV) moieties [12,17]. In particular, porphyrins and porphyrin-based compounds are some interesting ligands for organotin(IV) moieties, due not only to the property of the porphyrin to accumulate and to be retained by a variety of malignant lesions [18], but also their function as drug delivery systems [19], bio-sensing and bio-imaging molecules [20]. Indeed, the organotin(IV) complexes have different mechanisms of action on different cancer cells and induce tumour cell death in a concentration dependent manner [21,22]. In particular, organotin(IV) complexes at higher concentrations, induce cancer cell death, but at lower concentrations organotin(IV) complexes show antitumoral activity, inhibiting the cancer cell growth and therefore opening a new field of study in the metal-pharmaceutical research area [21,22]. Therefore, with the aim to identify new potential chemotherapeutic drugs that can inhibit the growth and metastatic dissemination of human melanoma cells, we studied the effects of melanoma treatment with two organotin(IV) compounds, namely the dibutyltin(IV) and tributyltin(IV) derivatives of *meso*-tetra(4-sulfonatophenyl)porphine, (Bu_2_Sn)_2_TPPS and (Bu_3_Sn)_4_TPPS. The complexes were synthesized as previously reported [8] and were isolated in the solid state as octahedral and trigonal-bipyramidal eq-R3Sn polymeric configurations, respectively, for (Bu_2_Sn)_2_TPPS and (Bu_3_Sn)_4_TPPS complexes, with the arylsulfonate groups behaving as monoanionic bidentate bridging ligands (Figure 1A,B).

We have previously reported the death due to apoptosis and in particular the activation of both the intrinsic and the extrinsic apoptotic pathways in human melanoma cells treated with micromolar concentrations of the (Bu_2_Sn)_2_TPPS and the (Bu_3_Sn)_4_TPPS [14,23,24,25]. In this paper we improved upon and expanded this field of research by identifying that nanomolar concentrations of (Bu_2_Sn)_2_TPPS and (Bu_3_Sn)_4_TPPS that are sufficient to significantly induce the cell cycle arrest of some melanoma cell lines through the inhibition of cyclin D1 expression.

We also reported that in melanoma cells (Bu_2_Sn)_2_TPPS and (Bu_3_Sn)_4_TPPS mediate inhibition of cell colony formation as well as of the integrin and CAM adhesion receptors expression and therefore, the inhibition of melanoma cell motility. Moreover, we showed that (Bu_2_Sn)_2_TPPS and (Bu_3_Sn)_4_TPPS act downstream of BRAF, mainly bypassing its functions but probably targeting STAT3 signalling protein.

In conclusion, the compounds (Bu_2_Sn)_2_TPPS and (Bu_3_Sn)_4_TPPS, for their role in the regression of the growth and migration of melanoma that we show in this paper, could deeply interfere with the melanoma progression and metastatic dissemination as new strategies for an effective treatment of this highly invasive tumour type.

## 2. Materials and Methods

### 2.1. Cell Lines, Antibodies, Chemicals and Reagents

The A375 (ATCC-CRL-1619) [26], HT-144 (ATCC-HTB-63) and M74 melanoma cell lines (kindly provided by Prof. C. Alcaide-Loridan, Institut Jacques Monod, Paris Diderot University, Paris, France) harbouring the BRAF V600E mutation, were grown in RPMI 1640 supplemented with 10% FCS and 1% penicillin-streptomycin (10,000 U/mL and 10,000 μg/mL, respectively) in 5% CO_2_ at 37 °C. The A375 cell line was isolated from a primary tumour on the skin and can form metastatic foci in nude mice [27]; the HT-144 cell line was isolated from a metastatic site on subcutaneous tissue [28] and the M74 cell line was established from metastatic tumour fragments [29]. The organotin(IV) *meso*-tetra(4-sulfonatophenyl)porphinate complexes, (Bu_2_Sn)_2_TPPS (formula weight FW = 1397) and (Bu_3_Sn)_4_TPPS (formula weight FW = 2091), were synthesized as previously reported [8] by refluxing, in dry methanol, Bu_2_SnO or (Bu_3_Sn)_2_O (gifts from Witco GmbH, Bergkamen, Germany), and *meso*-tetra(4-sulfonatophenyl)porphine (=H_4_TPPS) of high purity, obtained from Porphyrin Products (Logan, UT, USA). The (Bu_2_Sn)_2_TPPS and the (Bu_3_Sn)_4_TPPS were dissolved in dimethylsulfoxide (DMSO) to obtain a 200 μM stock solution before each treatment. Furthermore, the stock solutions were diluted in culture medium at various concentrations and before each treatment; in these solutions the final DMSO concentration did not exceed 0.05% (*v*/*v*). As a control, equal volumes of DMSO were added to untreated cells. The anti-pTyr-397 FAK motif, integrin β1, ICAM-1 and MCAM mouse monoclonal antibodies were obtained from BD Biosciences (Lexington, KY, USA). The anti-integrin β3, FAK and cyclin D1 rabbit polyclonal antibodies and the anti-BRAF mouse monoclonal antibody were obtained from Santa Cruz (Santa Cruz, CA, USA). The anti-PARP-1 rabbit polyclonal antibody was purchased from Cell Signaling Technology (Leiden, The Netherlands). The mouse monoclonal antibody direct against β-actin was obtained from Sigma (St Louis, MO, USA). The anti-mouse and anti-rabbit Alexa Fluor 680 conjugated and the infrared dye-conjugated IRDye800 anti-mouse and anti-rabbit were purchased as secondary antibodies from Molecular Probes (Eugene, OR, USA) and LI-COR Biosciences (Lincoln, NE, USA), respectively. All other chemicals were of analytical grade and were obtained from Sigma Chemical Co. (St Louis, MO, USA), Merck/VWR Readington, NJ, USA) or J. T. Baker (Phillipsburg, NJ, USA).

### 2.2. Cell Viability Assay (MTS Assay)

A375 (0.5 × 10^4^ per well) or HT-144 and M74 human melanoma cells (1 × 10^4^ per well) were seeded in 96-well plates for 24 h. Thereafter, the cells were treated with (Bu_2_Sn)_2_TPPS and (Bu_3_Sn)_4_TPPS or with DMSO as reported, for 24 h, 48 h and 72 h in triplicate and the cell viability was determined using the 3-(4,5-dimethylthiazol-2-yl)-5-(3-carboxymethoxyphenyl)-2-(4-sulfophenyl)-2*H*–tetrazolium (MTS) proliferation assay, following the instructions of the CellTiter-96 AQueous One Solution Cell Proliferation Assay (Promega, Madison, WI, USA). The plates were scanned at 490 nm in a 96-well plate reader after 30 min, 1 h, 1 h 30 min and 2 h. Cell viability for each treatment was normalized against DMSO treated cells. The concentrations of (Bu_2_Sn)_2_TPPS and (Bu_3_Sn)_4_TPPS required to achieve 50 % inhibition of the A375, HT-144 and M74 cell viability compared to untreated cell viability (IC_50_ values), were calculated after 24 h, 48 h and 72 h of treatment using a dose-response curve (Excel, Microsoft, Redmond, WA, USA).

### 2.3. Total Cell Extracts and Western Blot Analysis

Semi-confluent A375, HT-144 and M74 human melanoma cells were treated with (Bu_2_Sn)_2_TPPS and (Bu_3_Sn)_4_TPPS or with DMSO as reported, for 24 h, 48 h and 72 h. Therefore, the cells were lysed as previously reported [30], in particular we used RIPA buffer (50 mM Tris-HCl pH 8, 150 mM NaCl, 1% NP40, 0.5% DOC, 0.1% SDS) containing proteases and phosphatase inhibitors (4 mM PMSF and protease inhibitors cocktail, cocktail 2 and 3 of phosphatase inhibitors, Sigma, St Louis, MO, USA) for 30 min on ice. The cells in lysis buffer, were cleared of cellular debris by centrifugation at 12,000× *g* at 4 °C for 30 min. The Bradford protein assay (Bio-Rad laboratories GmbH, München, Germany) was used to determine the protein concentration of the supernatants. Forty μg of total cell extracts were resolved on sodium dodecyl sulfate-polyacrylamide gel electrophoresis (SDS-PAGE) and absorbed to nitrocellulose membrane (Hybond ECL, GE Healthcare Biosciences, Pittsburgh, PA, USA). After overnight incubation at 4 °C in blocking buffer (LI-COR Biosciences, Lincoln, NE, USA), the nitrocellulose membrane was incubated at room temperature for 1 h 30 min with primary antibodies and anti-mouse or anti-rabbit infrared fluorescent-labelled secondary antibodies (IRDye800, LI-COR Biosciences, Lincoln, NE, USA and Alexa Fluor 680 conjugated, Molecular Probes, Eugene, OR, USA) diluted in blocking solution (LI-COR Biosciences, Lincoln, NE, USA) and TBS-Tween buffer. The protein bands were scanned and the band intensities of each western blot were quantified by densitometric analysis, using the Odyssey infrared imaging system (LI-COR Biosciences, Lincoln, NE, USA). In the histograms are reported the mean values of several western blot experiments of almost three different experiments and are expressed as a per cent of unstimulated cells, normalized for the actin amount.

### 2.4. Cell Cycle Analysis

Semiconfluent A374, HT-144 and M74 melanoma cells treated for 72 h with (Bu_2_Sn)_2_TPPS and (Bu_3_Sn)_4_TPPS or with DMSO as reported, were washed twice with ice-cold PBS and resuspended at 1 × 10^6^ cells/ml in hypotonic fluorochrome solution (0.1% sodium citrate, 0.03% Nonidet P-40 and 50 µg/ml propidium iodide) for 30 min at room temperature in the dark. Therefore, the cells were acquired on a FACSCalibur™ flow cytometer, supported by CellQuest acquisition and data analysis software (Becton Dickinson, Mountain View, CA, USA).

### 2.5. Cell Colony Assay

A374, HT-144 and M74 melanoma cells (1.8 × 10^2^, 10.8 × 10^2^ and 18 × 10^2^ cells, respectively) were seeded in a 12-well cell culture plate and treated for 72 h with (Bu_2_Sn)_2_TPPS and (Bu_3_Sn)_4_TPPS or with DMSO as reported. Afterwards, the treated and untreated cells were maintained in fresh medium for 7–14 days, were fixed in 100% methanol and stained with 0.5% crystal violet in 20% methanol. Therefore, the plates were air dried, the colonies were photographed using a digital camera and counted.

### 2.6. Cell Migration Assay 

The A375 and HT-144 melanoma cells were treated as reported with (Bu_2_Sn)_2_TPPS and (Bu_3_Sn)_4_TPPS or with DMSO for 72 h. After treatment, as previously reported [30], 1.6 × 10^4^ of A375 and HT-144 melanoma cells were plated in serum-free medium in the inner chamber of a 24-well culture plate (Falcon, Bredford, MA, USA) with a polyethylene terephthalate (PET) membrane (pore size, 8 μm; Falcon, Bredford, MA, USA) [31]. The lower wells were filled with RPMI supplemented with 10% FBS. After 16 h at 37 °C the cells in the upper chamber were removed with a cotton swab and the migrated cells attached to the lower surface of the transwell membrane were fixed for 20 min with 100% methanol and stained for 1h with 0.5% crystal violet in 20% methanol. After staining, all the cells on the lower side of the filters were counted under phase contrast microscope (Leica, Wetzlar, Germany).

## 3. Results

### 3.1. Inhibition of Melanoma Cell Proliferation

**Figure 2:**
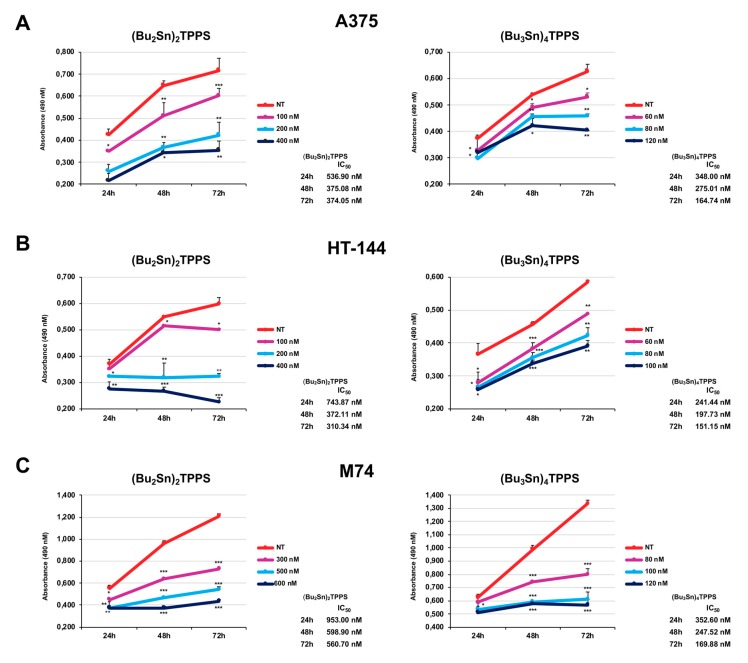
MTS assay of A375, HT-144 and M74 melanoma cells treated with (Bu_2_Sn)_2_TPPS and (Bu_3_Sn)_4_TPPS. The (**A**) A375 cells were treated with 100 nM, 200 nM and 400 nM of (Bu_2_Sn)_2_TPPS (left), or with 60 nM, 80 nM and 120 nM of (Bu_3_Sn)_4_TPPS (right); (**B**) the HT-144 cells were treated with 100 nM, 200 nM and 400 nM of (Bu_2_Sn)_2_TPPS (left), or with 60 nM, 80 nM and 100 nM of (Bu_3_Sn)_4_TPPS (right); (**C**) the M74 cells were treated with 300 nM, 500 nM and 600 nM of (Bu_2_Sn)_2_TPPS (left), or with 80 nM, 100 nM and 120 nM of (Bu_3_Sn)_4_TPPS (right) for 24 h, 48 h and 72 h and the cell proliferation rate of untreated (NT) and treated melanoma cells, was analysed through MTS assay. In the histograms (**A**–**C**) are reported the mean values of almost three different experiments performed in triplicate. The error bars indicate the standard deviation, statistical significance was analysed by the Student’s *t*-test: *p < 0.05 was considered significant; **p < 0.01 highly significant; ***p < 0.001 very highly significant. The IC_50_ values are also reported using the values of three different experiments performed in triplicate.

With the aim of identifying the minimum concentrations of (Bu_2_Sn)_2_TPPS and (Bu_3_Sn)_4_TPPS (Figure 1A,B) sufficient to inhibit the growth of the A375, HT-144 and M74 human melanoma cell lines, we analysed the cellular growth of melanoma cells treated with (Bu_2_Sn)_2_TPPS concentrations ranging from 100 nM to 600 nM and with (Bu_3_Sn)_4_TPPS ranging from 60 nM to 120 nM, for 24 h, 48 h and 72 h, through MTS assays (Figure 2). The results of these experiments showed that the treatment of A375 and HT-144 cells with 200 nM and of M74 cells with 300 nM of (Bu_2_Sn)_2_TPPS and the treatment of A375, HT-144 and M74 cells with 80 nM, 60 nM and 100 nM of (Bu_3_Sn)_4_TPPS, respectively, induce the inhibition (Figure 2A, right panel, Figure 2B left panel and Figure 2C, left and right panels) or the decrease (Figure 2A, left panel and Figure 2B, right panel) of the cell proliferation to a greater extent after 48–72 h of treatment, compared to untreated cells (NT). Interestingly, the IC_50_ values obtained for both compounds are higher than the amounts required to inhibit or reduce the cell proliferation (Figure 2). The A375, the HT-144 and the M74 human melanoma cell lines express the mutated form V600E of BRAF, in the A375 and in the M74 cells the promoter of the telomerase reverse transcriptase (TERT) is mutated and in M74 cells the expression of p53 is very low, furthermore in the HT-144 cells also the Ataxia Telangiectasia Mutated (ATM) protein is mutated [32,33,34]. All these mutations could induce a different growth rate of melanoma cells and also different sensitivity to the treatment with (Bu_2_Sn)_2_TPPS and with (Bu_3_Sn)_4_TPPS compounds. In particular, ATM is activated by DNA double-strand breaks (DSB), is involved in cell-cycle control, DSB repair and in the block of the cell cycle progression [35]. Therefore, the mutation of ATM in HT-144 cells could slow down the response to the (Bu_3_Sn)_4_TPPS compound used in these experiments that probably, for its high DNA binding affinity, induces DNA damage in melanoma cells. Furthermore, in A375 and M74 melanoma cells, the mutation of TERT promoter, could be associated with increased TERT expression and cell survival [32].

**Figure 3:**
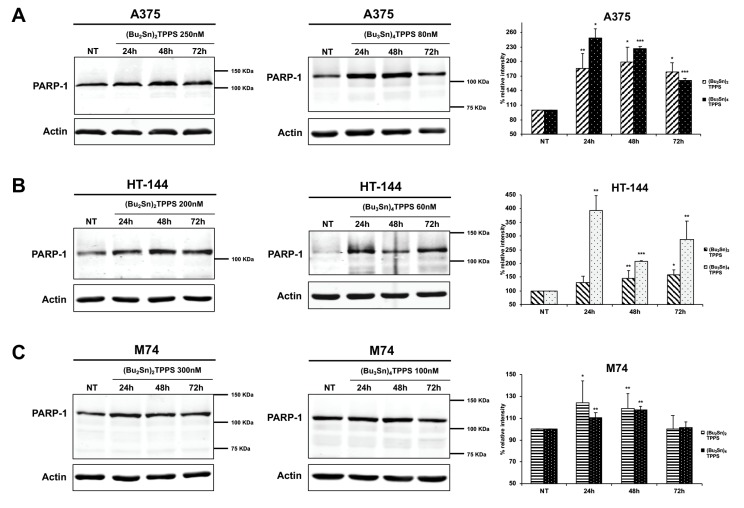
Kinetics of PARP-1 expression in A375, HT-144 and M74 melanoma cells treated with (Bu_2_Sn)_2_TPPS and (Bu_3_Sn)_4_TPPS. The kinetics of PARP-1 expression was analysed through western blot experiments in (**A**) A375, (**B**) HT-144 and (**C**) M74 cell lines in response to the treatment with 250 nM, 200 nM and 300 nM of (Bu_2_Sn)_2_TPPS (left and right panels) and with 80 nM, 60 nM and 100 nM of (Bu_3_Sn)_4_TPPS respectively (middle and right panels) for 24 h, 48 h and 72 h. The analysis of the β-actin expression (**A**, **B** and **C**, lower panels) was used to confirm the equal protein loading. The error bars indicate the standard deviation. The Student’s t-test was used to the analysis of statistical significance: *p < 0.05 was considered significant; **p < 0.01 highly significant; ***p < 0.001 very highly significant (**A**, **B** and **C**, right panels).

Furthermore, in the light of the results of the MTS assay, we treated the A375, HT-144 and M74 melanoma cells for 24 h, 48 h and 72 h with 250 nM, 200 nM and 300 nM, respectively, of (Bu_2_Sn)_2_TPPS and with 80 nM, 60 nM and 100 nM of (Bu_3_Sn)_4_TPPS, to study the consequences of the melanoma treatment with low concentrations of the complexes. Indeed, in the following experiments we treated the A375 cells with 250 nM of (Bu_2_Sn)_2_TPPS instead of the 200 nM used in MTS assay sufficient only to decrease the A375 proliferation rate. In particular, we analysed through western blot experiments, the expression of the poly (ADP-ribose) polymerase (PARP-1) (Figure 3), using an antibody direct against the full-length and the cleaved forms of PARP-1. Indeed, PARP-1 is a nuclear enzyme that plays a pivotal role in many processes such as the repair of DNA single and double strand breaks [36,37] and its cleavage is an apoptotic marker and an indicator of caspase activation [38]. Therefore, we showed that the treatment with (Bu_2_Sn)_2_TPPS (Figure 3A–C, left and right panels) and with (Bu_3_Sn)_4_TPPS (Figure 3A–C, middle and right panels), led to a significant increase of the full-length form of PARP-1 that is more evident in A375 and HT-144 melanoma cells treated with (Bu_3_Sn)_4_TPPS (Figure 3A,B, middle and right panels), but do not induce the cleavage of PARP-1 in treated melanoma cells (Figure 3A–C). Therefore, the increase of full-length form of PARP-1 but the lack of PARP-1 cleavage, strongly suggest that these treatments induce DNA damage but do not induce the death for apoptosis of treated melanoma cells.

**Figure 4:**
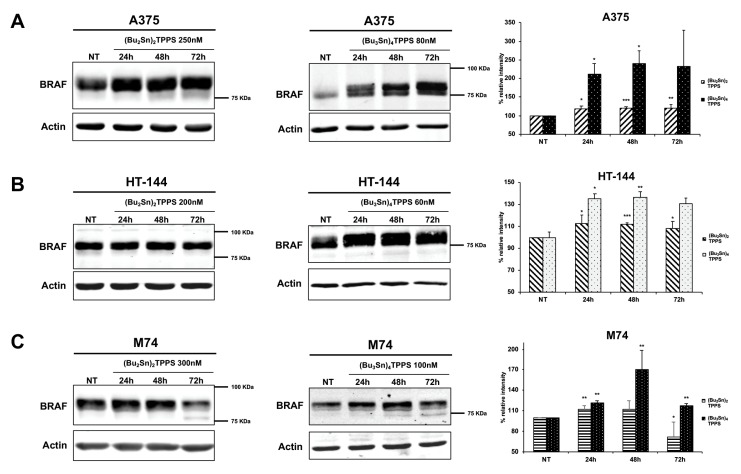
Kinetics of BRAF expression in A375, HT-144 and M74 melanoma cells treated with (Bu_2_Sn)_2_TPPS and (Bu_3_Sn)_4_TPPS. The kinetics of BRAF expression was analysed in (**A**) A375, (**B**) HT-144 and (**C**) M74 cell lines treated with 250 nM, 200 nM and 300 nM of (Bu_2_Sn)_2_TPPS (left and right panels) and with 80 nM, 60 nM and 100 nM of (Bu_3_Sn)_4_TPPS (middle and right panels) respectively, for 24 h, 48 h and 72 h through western blot experiments. The analysis of the β-actin expression (**A**, **B** and **C**, lower panels) was used to confirm the equal protein loading. The error bars indicate the standard deviation. The Student’s *t*-test was used to the analysis of statistical significance: *p < 0.05 was considered significant; **p < 0.01 highly significant; ***p < 0.001 very highly significant (**A**, **B** and **C**, right panels).

Furthermore, we analysed through western blot experiments, the expression of BRAF (Figure 4), a key signalling protein which plays a critical role in the regulation of cellular growth and proliferation [39]. In particular, we showed the increased expression of BRAF in treated cells compared to untreated cells (NT), that is more evident in melanoma cells treated with (Bu_3_Sn)_4_TPPS (Figure 4A–C, middle and right panels). Therefore, the inhibition of the cellular proliferation but the increase of the BRAF expression, strongly suggest that these treatments can bypass some of the BRAF functions.

### 3.2. Cell Cycle Arrest of Treated Melanoma Cells

**Figure 5:**
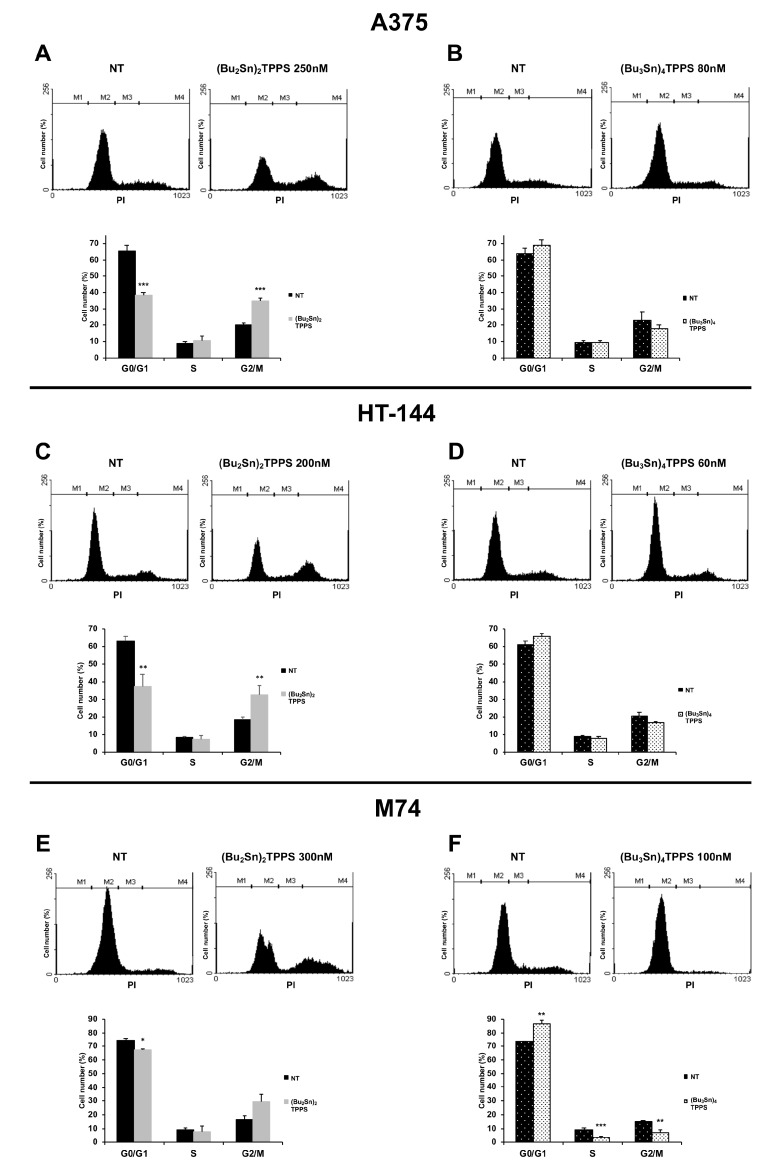
Cell-cycle analysis of A375, HT-144 and M74 melanoma cells treated with nanomolar concentration of (Bu_2_Sn)_2_TPPS and (Bu_3_Sn)_4_TPPS. Twenty thousand cells (events) per sample of untreated (NT) or treated for 72 h, A375 (**A**,**B**), HT-144 (**C**,**D**) and M74 (**E**,**F**) melanoma cells with (**A**) 250 nM, (**C**) 200 nM, (**E**) 300 nM of (Bu_2_Sn)_2_TPPS and with (**B**) 80 nM, (**D**) 60 nM, (**F**) 100 nM of (Bu_3_Sn)_4_TPPS respectively, were stained with PI. The fluorescence of PI-stained cells was analysed through flow cytometry to identify the percentage rate of cells in G0/G1, S and G2/M phases of the cell cycle. In the histograms (**A**–**F**, lower panels) the cell fractions of treated cells in G0/G1, S and G2/M phase of the cell cycle are expressed as a per cent of the cell fractions of untreated cells and as mean values of almost three different experiments, the error bars indicate the standard deviation and statistical significance was analysed by the Student’s *t*-test: *p < 0.05 was considered significant; **p < 0.01 highly significant; ***p < 0.001 very highly significant.

The highly conserved DNA-repair and cell cycle checkpoint pathways, are some mechanisms activated by the cells in response to DNA damage, to delay the cell cycle progression, to identify errors and to efficiently repair the damaged DNA [40]. Therefore, with the aim to further investigate the mechanisms used by these compounds to inhibit the cellular growth, we treated the A375, HT-144 and M74 cells with respectively 250 nM, 200 nM and 300 nM of (Bu_2_Sn)_2_TPPS and with 80 nM, 60 nM and 100 nM of (Bu_3_Sn)_4_TPPS for 72 h and the cell cycle distribution of treated and untreated melanoma cells was analysed by flow cytometry (Figure 5). In particular, we showed that the (Bu_2_Sn)_2_TPPS treatment decreases the rate of A375, HT-144 and M74 melanoma cells in G0/G1 phase (38.50%, 37.43% and 67.39%) compared to untreated cells (NT) (65.69%, 63.07% and 74.26%), but increases the rate of the cells in G2/M phase (34.98%, 32.61% and 29.72%) compared to untreated cells (NT) (20.46%, 18.44% and 16.81%) (Figure 5A,C,E). Otherwise, the (Bu_3_Sn)_4_TPPS treatment increases the rate of A375, HT-144 and M74 melanoma cells in G0/G1 phase (68.88%, 65.96% and 86.49%) compared to untreated cells (NT) (63.68%, 61.44% and 73.41%), but decreases the rate of the cells in G2/M phase (18.11%, 16.52% and 7.01%) compared to untreated cells (NT) (23.02%, 20.39% and 14.81%), (Figure 5B,D,F). Therefore, the results of these experiments show that (Bu_2_Sn)_2_TPPS and the (Bu_3_Sn)_4_TPPS exert their anti-proliferative effects in melanoma cells through the blockage of cell cycle progression using different mechanisms (Figure 5).

**Figure 6:**
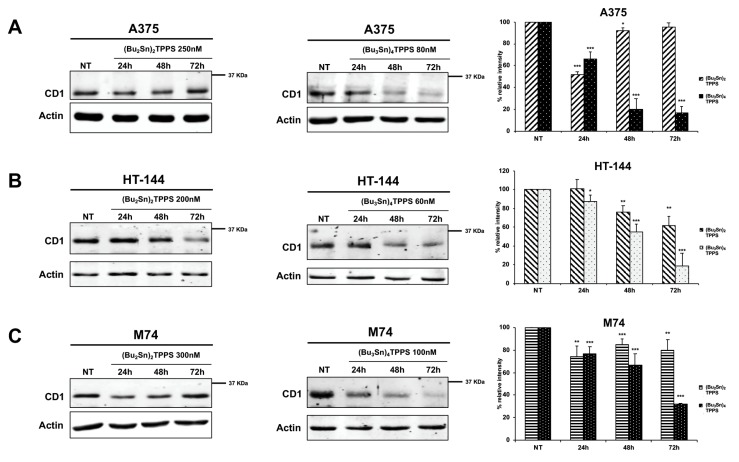
Kinetics of cyclin D1 expression in A375, HT-144 and M74 melanoma cells treated with (Bu_2_Sn)_2_TPPS and (Bu_3_Sn)_4_TPPS. The kinetics of cyclin D1 (CD1) expression was analysed in (**A**) A375, (**B**) HT-144 and (**C**) M74 cell lines treated with 250 nM, 200 nM and 300 nM of (Bu_2_Sn)_2_TPPS (left and right panels) and with 80 nM, 60 nM and 100 nM of (Bu_3_Sn)_4_TPPS (middle and right panels) respectively, for 24 h, 48 h and 72 h, through western blot experiments. The analysis of the β-actin expression (**A**, **B** and **C**, lower panels) was used to confirm the equal protein loading. The error bars indicate the standard deviation. The Student’s t-test was used to the analysis of statistical significance: *p < 0.05 was considered significant; **p < 0.01 highly significant; ***p < 0.001 very highly significant (**A**, **B** and **C**, right panels).

Interestingly, each stage of the cell cycle is regulated by different complexes of the cyclin dependent kinases (CDKs) and of their regulatory partners, the cyclins [41]. Therefore, the expression of cyclin D1, a key regulator mainly of the G1/S [42] but also of the G2/M phases, was analysed by western blot experiments (Figure 6) on A375, HT-144 and M74 melanoma cells treated as reported for 24 h, 48 h and 72 h with (Bu_2_Sn)_2_TPPS and with (Bu_3_Sn)_4_TPPS. We showed that, as expected, a larger decrease of the cyclin D1 expression in (Bu_3_Sn)_4_TPPS-treated melanoma cells blocked in the G0/G1 phase (Figure 6A–C, middle and right panels) compared to the cells treated with (Bu_2_Sn)_2_TPPS and blocked in G2/M phase (Figure 6A–C, left and right panels). Interestingly, these results suggest that the treatment with nanomolar concentration of (Bu_2_Sn)_2_TPPS and (Bu_3_Sn)_4_TPPS induce G2/M and the G0/G1 cell cycle arrest, respectively, through the inhibition of cyclin D1 expression, although with different quantitative effects (Figure 6).

### 3.3. Cell Colony Formation 

**Figure 7:**
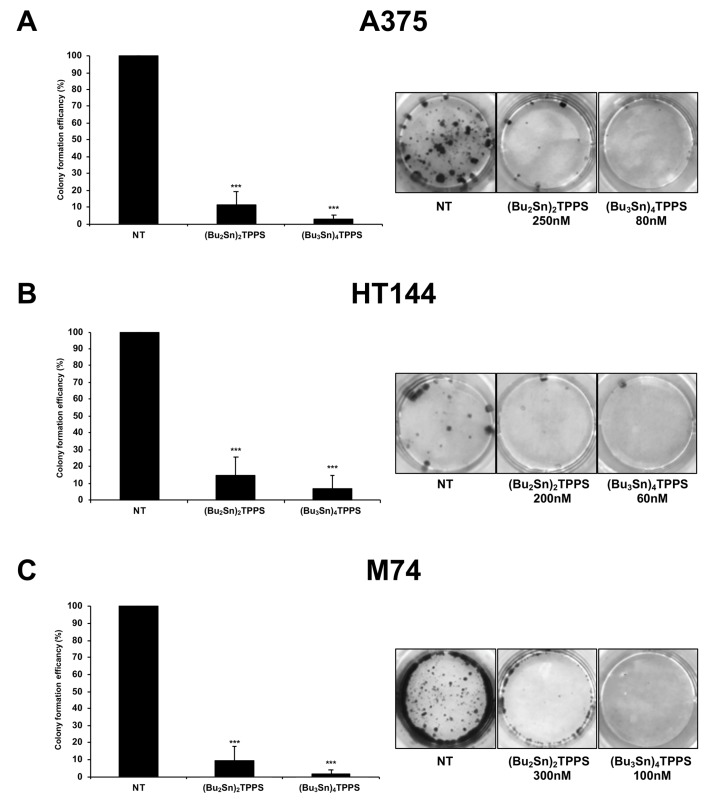
Cell colony assay of A375, HT-144 and M74 melanoma cells treated with nanomolar concentration of (Bu_2_Sn)_2_TPPS and (Bu_3_Sn)_4_TPPS. The A375 (**A**), the HT-144 (**B**) and the M74 (**C**) melanoma cells were treated for 72h with 250, 200 and 300 nM of (Bu_2_Sn)_2_TPPS (left) and with 80, 60 and 100 nM of (Bu_3_Sn)_4_TPPS (right), respectively. After 7–14 days of growth in fresh medium, the colonies of treated and untreated cells were fixed, stained, photographed using a digital camera and counted. The colony formation efficiency of treated cells is the number of the colonies formed by treated cells expressed as a per cent of colonies formed by untreated cells (NT). These data are reported in the histograms (**A**–**C**, left panels) as the mean values of almost three different experiments performed in triplicate. The error bars indicate the standard deviation and statistical significance was analysed by the Student’s *t*-test: ***p < 0.001 very highly significant.

The hallmark of chemotherapeutic drugs is the inhibition of cellular proliferation as well as the reduction of cell viability. Therefore, the ability of the A375, HT-144 and M74 cell lines to form colonies after treatment for 72 h with (Bu_2_Sn)_2_TPPS (250 nM, 200 nM and 300 nM, respectively) and with (Bu_3_Sn)_4_TPPS (80 nM, 60 nM and 100 nM, respectively) was analysed (Figure 7). Interestingly, the (Bu_2_Sn)_2_TPPS and the (Bu_3_Sn)_4_TPPS treatments highly inhibited the ability of melanoma cells to form colonies, in particular the A375, HT-144 and M74 cell lines treated with (Bu_2_Sn)_2_TPPS, displayed 11.44%, 14.52% and 9.60%, respectively, while the A375, HT-144 and M74 cell lines treated with (Bu_3_Sn)_4_TPPS, displayed 2.63%, 6.71% and 1.62%, respectively, of cell colony formation activity compared to untreated cells (Figure 7A–C). Therefore, our results showed that the treatment with nanomolar concentrations of (Bu_2_Sn)_2_TPPS and (Bu_3_Sn)_4_TPPS reduces the cell viability and the survival rate of melanoma cells.

### 3.4. Expression of Adhesion Receptors and FAK Signalling Protein 

**Figure 8:**
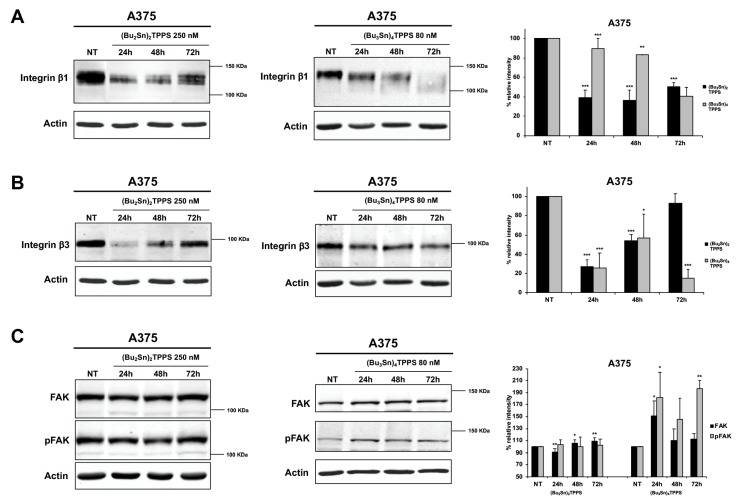
Kinetics of the integrins and FAK expression in A375 cells treated with (Bu_2_Sn)_2_TPPS and (Bu_3_Sn)_4_TPPS. The kinetics of (**A**) integrin β1, (**B**) integrin β3 adhesion receptors and of (**C**) FAK and pFAK expression was analysed in A375 melanoma cells treated with 250 nM of (Bu_2_Sn)_2_TPPS (A, B and C, left and right panels) and with 80 nM of (Bu_3_Sn)_4_TPPS (**A**, **B** and **C**, middle and right panels) for 24 h, 48 h and 72 h, through western blot experiments. The analysis of the β-actin expression (**A**, **B** and **C**, lower panels) was used to confirm the equal protein loading. The error bars indicate the standard deviation. The Student’s *t*-test was used to the analysis of statistical significance: *p < 0.05 was considered significant; **p < 0.01 highly significant; ***p < 0.001 very highly significant (**A**, **B** and **C**, right panels).

**Figure 9:**
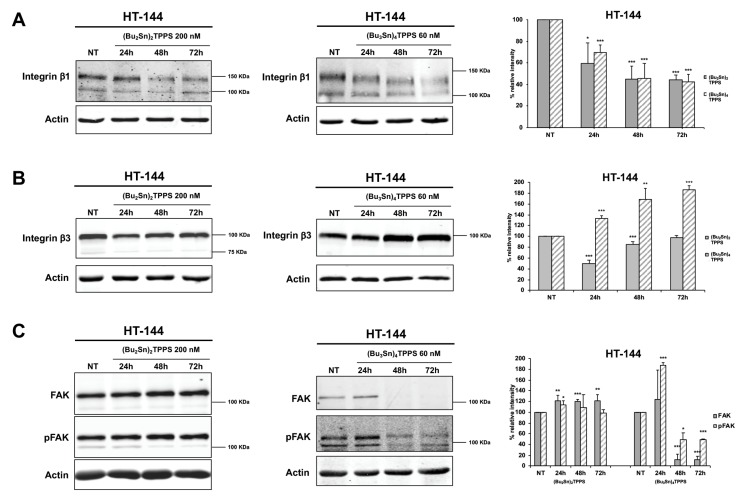
Kinetics of the integrins and FAK expression in HT-144 cells treated with (Bu_2_Sn)_2_TPPS and (Bu_3_Sn)_4_TPPS. The kinetics of (**A**) integrin β1, (**B**) integrin β3 adhesion receptors and of (**C**) FAK and pFAK expression was analysed in HT-144 melanoma cells treated with 200 nM of (Bu_2_Sn)_2_TPPS (**A**, **B** and **C**, left and right panels) and with 60 nM of (Bu_3_Sn)_4_TPPS (**A**, **B** and **C**, middle and right panels) for 24 h, 48 h and 72 h, through western blot experiments. The analysis of the β-actin expression (**A**, **B** and **C**, lower panels) was used to confirm the equal protein loading. The error bars indicate the standard deviation. The Student’s *t*-test was used to the analysis of statistical significance: *p < 0.05 was considered significant; **p < 0.01 highly significant; ***p < 0.001 very highly significant (**A**, **B** and **C**, right panels).

**Figure 10:**
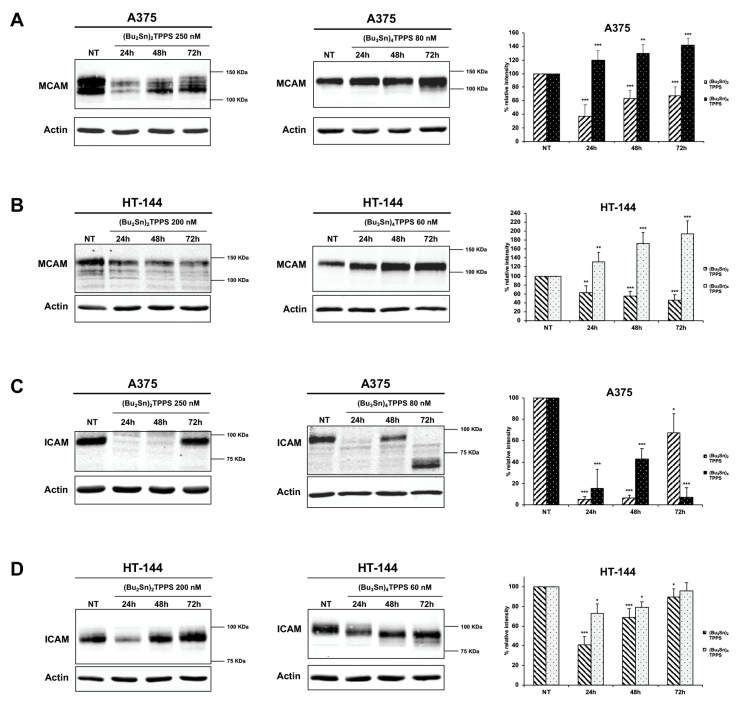
Kinetics of the CAMs expression in A375 and HT-144 cells treated with (Bu_2_Sn)_2_TPPS and (Bu_3_Sn)_4_TPPS. The kinetics of MCAM (**A**,**B**) and ICAM (**C**,**D**) expression was analysed in (**A**,**C**) A375 and in (**B**,**D**) HT-144 cell lines in response to the treatment with 250 nM and 200 nM of (Bu_2_Sn)_2_TPPS (left and right panels) and with 80 nM and 60 nM of (Bu_3_Sn)_4_TPPS (middle and right panels) respectively, for 24 h, 48 h and 72 h, through western blot experiments. The analysis of the β-actin expression (**A**, **B**, **C** and **D**, lower panels) was used to confirm the equal protein loading. The error bars indicate the standard deviation. The Student’s t-test was used to the analysis of statistical significance: *p < 0.05 was considered significant; **p < 0.01 highly significant; ***p < 0.001 very highly significant (**A**, **B**, **C** and **D**, right panels).

The adhesion receptors are important actors in melanoma metastatic dissemination defining the cellular shape and regulating cell mobility and invasiveness [3,43]. Therefore, to better understand the mechanisms used by (Bu_2_Sn)_2_TPPS and (Bu_3_Sn)_4_TPPS to inhibit the metastatic dissemination of melanoma cells, we analysed the expression of integrin β1 and β3 (Figure 8 and Figure 9), MCAM and ICAM (Figure 10) adhesion receptors in A375 (Figure 8, Figure 10A,C) and HT-144 (Figure 9, Figure 10B,D) cell lines treated for 24 h, 48 h and 72 h with 250 and 200 nM of (Bu_2_Sn)_2_TPPS, respectively, and with 80 and 60 nM of (Bu_3_Sn)_4_TPPS, respectively. In particular we showed in A375 and HT-144 melanoma cells treated with (Bu_2_Sn)_2_TPPS, the decreased expression of integrin β1 (Figure 8A and Figure 9A, left and right panels), integrin β3 (Figure 8B and Figure 9B, left and right panels), MCAM (Figure 10A,B, left and right panels) and ICAM (Figure 10C,D, left and right panels) adhesion receptors, with the exception of the steady state level expression of integrin β3 after 72 h of treatment in both cell lines (Figure 8B and Figure 9B, left and right panels), compared to untreated cells (NT).

The (Bu_3_Sn)_4_TPPS treatment induces the decreased expression of integrin β1 in both cell lines (Figure 8A and Figure 9A, middle and right panels), while the expression of integrin β3 decreased in A375 cells (Figure 8B, middle and right panels) but increased in HT-144 cells (Figure 9B, middle and right panels), compared to untreated cells (NT).

Finally, the (Bu_3_Sn)_4_TPPS treatment induces in both cell lines the increased expression of MCAM (Figure 10A,B, middle and right panels) as well as the decreased expression of ICAM (Figure 10C,D, middle and right panels) with the exception of the steady state level expression of ICAM after 72 h of treatment in HT-144 cells (Figure 10D, middle and right panels).

Integrin signalling leads to the activation of FAK through the auto-phosphorylation of the Tyr-397 [44], therefore, we analysed in treated and untreated (NT) melanoma cells, the expression and the activation of FAK signalling protein (Figure 8C and Figure 9C) and we showed in (Bu_2_Sn)_2_TPPS-treated cells that the expression and the activation of FAK are almost at the steady state level in both cell lines (Figure 8C and Figure 9C, left and right panels). Furthermore, we showed that the FAK expression and activation increase in (Bu_3_Sn)_4_TPPS treated A375 cells, (Figure 8C, middle and right panels), but decrease in HT-144 cells treated with (Bu_3_Sn)_4_TPPS for 48 h and 72 h, (Figure 9C, middle and right panels). In conclusion, these results collectively showed that the treatment with nanomolar concentrations of (Bu_2_Sn)_2_TPPS and (Bu_3_Sn)_4_TPPS induces in melanoma cells, although with different quantitative effects, the decreased expression of integrin and CAM adhesion receptors and in HT-144 cells treated with (Bu_3_Sn)_4_TPPS, the decreased expression and activation of FAK, thus suggesting that these complexes could inhibit the migration and metastatic dissemination of melanoma cells using different mechanisms.

### 3.5. Analysis of Melanoma Cell Migration

**Figure 11:**
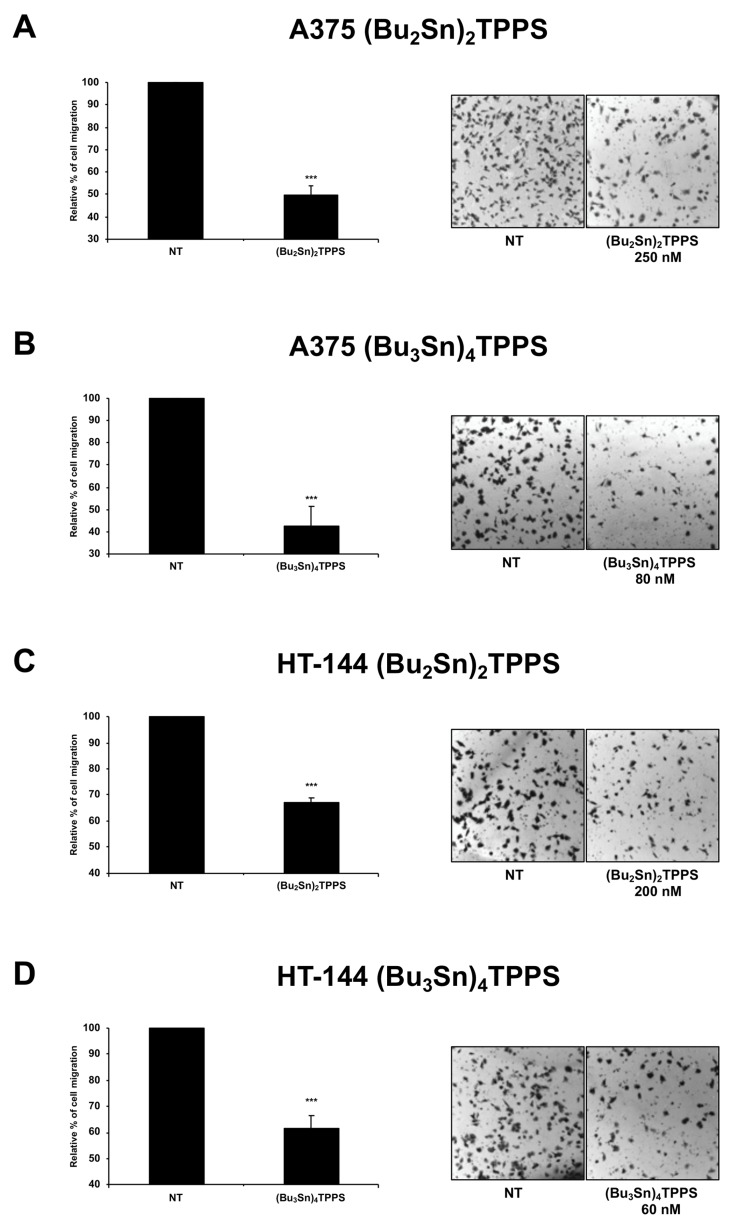
The treatment with nanomolar concentrations of (Bu_2_Sn)_2_TPPS and (Bu_3_Sn)_4_TPPS inhibit the migration of A375 and HT-144 melanoma cells. The (**A**,**B**) A375 and (**C**,**D**) HT-144 melanoma cells untreated (NT) or treated for 72 h with (**A**) 250 nM, (**C**) 200 nM of (Bu_2_Sn)_2_TPPS and with (**B**) 80 nM, (**D**) 60 nM of (Bu_3_Sn)_4_TPPS respectively, were plated in the inner chamber of a 24-well culture plate with PET membrane (8 μm pore size) and incubated at 37 °C for 16 h. The migrated cells fixed and stained, were counted under phase contrast microscope (Leica). The number of treated migrating cells, are expressed as a per cent of the untreated migrating cells and reported in the histograms (**A**–**D**, left panels) as the mean values of almost three different experiments performed in triplicate. The error bars indicate the standard deviation and statistical significance was analysed by the Student’s *t*-test: *p < 0.05 was considered significant; **p < 0.01 highly significant; ***p < 0.001 very highly significant.

Indeed, a complex network of adhesion receptors and signalling molecules are involved in the multistep process required to manage the cell migration, that play a key role on the aggressive metastatic trend of melanoma [43]. Therefore, in A375 and HT-144 cell lines treated for 72 h with 250 nM and 200 nM of (Bu_2_Sn)_2_TPPS, respectively, and with 80 nM and 60 nM of (Bu_3_Sn)_4_TPPS, respectively, we analysed the cellular migration ability (Figure 11). In particular, through the migration assay, we showed that the treatment for 72 h with (Bu_2_Sn)_2_TPPS and with (Bu_3_Sn)_4_TPPS decreases the migration ability of A375 cells (by 50.27% and 57.27%, respectively) (Figure 11A,B) and of HT-144 cells (by 32.8% and 38.2%, respectively) (Figure 11C,D), compared to untreated cells (NT). In conclusion, our results show that (Bu_2_Sn)_2_TPPS and (Bu_3_Sn)_4_TPPS treatment inhibit the migration and the spreading of melanoma cells, thus suggesting that could play a main role on the treatment of metastatic melanoma.

### 3.6. Expression of STAT3 Signalling Protein 

**Figure 12:**
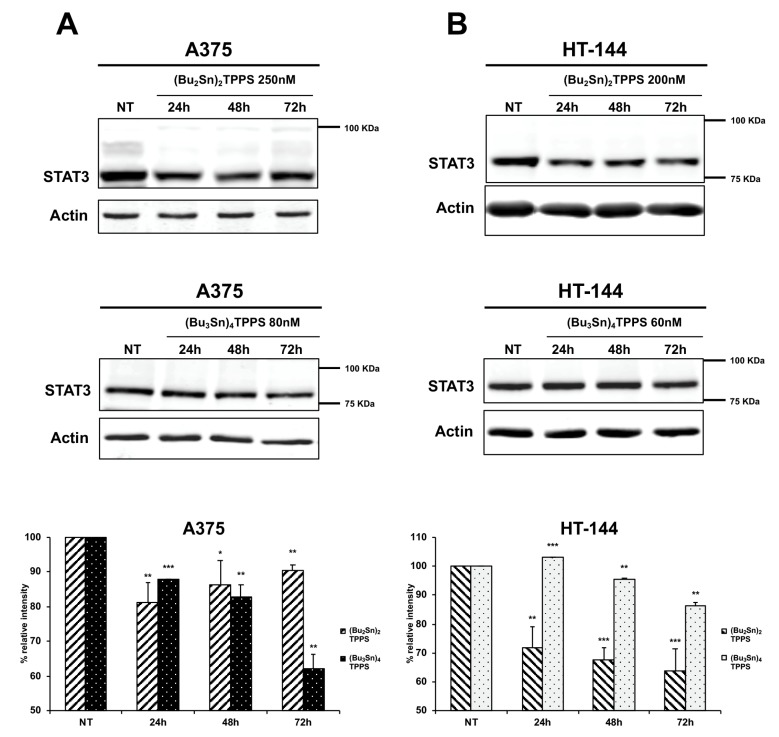
Kinetics of STAT3 expression in A375 and HT-144 melanoma cells treated with (Bu_2_Sn)_2_TPPS and (Bu_3_Sn)_4_TPPS. The kinetics of STAT3 expression was analysed in (**A**) A375 and (**B**) HT-144 cell lines in response to the treatment with 250 nM and 200 nM of (Bu_2_Sn)_2_TPPS (**A** and **B**, higher and lower panels) and with 80 nM and 60 nM of (Bu_3_Sn)_4_TPPS (**A** and **B**, middle and lower panels) respectively, for 24 h, 48 h and 72 h, through western blot experiments. The analysis of the β-actin expression (**A**,**B**) was used to confirm the equal protein loading. The error bars indicate the standard deviation. The Student’s *t*-test was used to the analysis of statistical significance: *p < 0.05 was considered significant; **p < 0.01 highly significant; ***p < 0.001 very highly significant (**A** and **B**, lower panels).

The STAT3 protein is often constitutively activated in melanoma cells and its inhibition in melanoma induces cell death, tumour regression and the inhibition of metastatic dissemination [45,46]. Therefore, in A375 (Figure 12A) and in HT-144 (Figure 12B) cell lines treated for 24 h, 48 h and 72 h with (Bu_2_Sn)_2_TPPS (250 nM and 200 nM, respectively) and with (Bu_3_Sn)_4_TPPS (80 nM and 60 nM, respectively), we analysed through western blot the expression of STAT3 signalling protein (Figure 12). In particular, we showed in both cell lines treated with (Bu_2_Sn)_2_TPPS (Figure 12A,B, upper and lower panels) and with (Bu_3_Sn)_4_TPPS (Figure 12A,B, middle and lower panels), the decreased expression of STAT3 compared to untreated cells (NT). In conclusion, these results indicate that in melanoma cells, the STAT3 signalling protein is a target of the (Bu_2_Sn)_2_TPPS and (Bu_3_Sn)_4_TPPS that could mediate the inhibition of melanoma proliferation and metastatic dissemination weakened by these complexes.

## 4. Discussion

Melanoma is one of the most treatment-refractory malignancies, notorious for its propensity to metastasize. Indeed, notwithstanding the effectiveness of new therapeutic approaches such as immunotherapies and targeted therapies [4], the melanoma field is in great need to identify new therapeutic approaches. Therefore, to improve the research of potential anticancer drugs, we studied the effects of the treatment with nanomolar concentrations of two organotin(IV) complexes of *meso*-tetra(4-sulfonatophenyl)porphine, (Bu_2_Sn)_2_TPPS and (Bu_3_Sn)_4_TPPS, on the growth and on the metastatic dissemination of some human melanoma cell lines. In particular, we showed the induction of a dose-dependent inhibition of cellular growth and respectively the G2/M and the G0/G1 cell cycle arrest, the increased expression of the full-length PARP-1 as well as the inhibition of the cell colony formation and cell migration. PARP-1(ADP-ribosyltransferase-1), a member of the poly-ADP-ribose polymerase family, is involved in many different processes such as DNA single and double strand break repair [47], chromatin modification, transcriptional regulation and cell death [36]. The activation of PARP-1 follows his binding to the DNA strand breaks and enables PARP-1 to synthetize poly(ADP-ribose) (PAR) and also to increase its own expression [47]. Therefore, the hypothesis that the melanoma cells treatment with nanomolar concentrations of (Bu_2_Sn)_2_TPPS and (Bu_3_Sn)_4_TPPS induces DNA damage, elicits the increased expression of PARP-1 that, however, is not sufficient to repair an overwhelming amount of DNA damage and to avoid the consequent block of cell cycle. However, in treated M74 cells we reported a lower increase of PARP-1 expression compared to the expression level of PARP-1 in A375 and HT-144 treated cells, probably due to the low expression in M74 cells, of a PARP-1-activating protein such as the p53 protein [34,48]. Furthermore, the A375, HT-144 and M74 human melanoma cell lines harbouring the mutation V600E of BRAF, also express the mutated form of several common and different proteins such as ATM in HT-144 cells, instead, in A375 and M74 cells the promoter of TERT is mutated [32,33,34], thus eliciting the reported different sensitivity to the treatment with (Bu_2_Sn)_2_TPPS and (Bu_3_Sn)_4_TPPS. Indeed, the hydrophobic butyl components of the (Bu_2_Sn)_2_TPPS and the (Bu_3_Sn)_4_TPPS, can directly interact with DNA and in particular, the tin of the organotin(IV) compounds can bind not only to the DNA bases but also the functional groups of the DNA grooves and the phosphate of the DNA phosphodiester backbones [11,12,13,15]. Therefore, the high DNA binding affinity of these organotin(IV) compounds and mostly of (Bu_3_Sn)_4_TPPS, elicits the alteration of the DNA conformation [15] and the induction of DNA damage suggested by the increased expression of the full-length PARP-1 as well as by the inhibition of the growth and by the blockage of the cell cycle that we showed in treated melanoma cells. In particular, our hypothesis is that (Bu_3_Sn)_4_TPPS has a higher DNA backbone affinity compared to (Bu_2_Sn)_2_TPPS and therefore the higher alteration of the DNA conformation could explain the earlier stop of the cell cycle reported in (Bu_3_Sn)_4_TPPS treated cells. Interestingly, we also showed in treated melanoma cells the decreased expression of the protein cyclin D1, a key regulator of the G1/S but also of the G2/M phase transition [42]. Moreover, cyclin D1 can promote cancer formation and cancer survival through the regulation of transcription, DNA damage and repair, the induction of chromosomal instability as well as the enhancement of angiogenesis, cell migration and invasion [49,50]. Therefore, in the light of the polyhedral functions of cyclin D1, the inhibition of cancer growth and metastatic diffusion induced by these compounds in melanoma cells, could be also mediated by the inhibition of cyclin D1 expression. Furthermore, the treatment of melanoma cells carrying the BRAF mutation V600E with nanomolar concentrations of (Bu_2_Sn)_2_TPPS and (Bu_3_Sn)_4_TPPS induces the increased expression of BRAF, notwithstanding the fact these treatments also induce the inhibition of cellular growth, the blockage of the cell cycle, the inhibition of cell colony formation and the inhibition of the expression of a BRAF target such as cyclin D1 [51]. BRAF is a serine-threonine protein kinase mutated in almost the 50%–60% of melanomas [2] mostly harbouring the mutation V600E that increases the protein kinase activity, the consequent activation of the BRAF/MAPK/ERK signalling pathways and therefore the tumour cell growth and proliferation [39]. Therefore, the reported results show that the downstream functions of BRAF, such as melanoma growth, mobility and invasiveness are irreparably compromised, suggest that (Bu_2_Sn)_2_TPPS and (Bu_3_Sn)_4_TPPS act downstream of BRAF mainly bypassing its functions. Indeed, these results make it appealing to follow up the studies of the signalling pathways inhibited by (Bu_2_Sn)_2_TPPS and (Bu_3_Sn)_4_TPPS treatment as very interesting therapeutic strategies. Melanoma can metastasize spreading beyond the primary tumour and this aggressive metastatic trend is mediated by some adhesion receptors that promote the cell migration during each phase of cancer development and progression but also promote the metastatic dissemination of cancer cells [3,43]. In melanoma cells the treatment with nanomolar concentrations of (Bu_2_Sn)_2_TPPS and (Bu_3_Sn)_4_TPPS is mostly associated, although with different outcomes, to a significant decrease of the expression of integrin and CAM adhesion receptors and especially to the consequent inhibition of the cellular motility. The lipophilicity of the butyl moieties [15] enables (Bu_2_Sn)_2_TPPS and (Bu_3_Sn)_4_TPPS to cross and to intercalate the lipid bilayer of the membrane, to bind phospholipids, glycoproteins and receptors, thus modifying the structure and functionality of the membranes [52], could explain the reported results. Therefore, the decreased expression of integrin and CAM adhesion receptors in treated melanoma cells, could inhibit not only the cellular motility, but also the homologous and heterologous interactions between melanoma and endothelial cells thus preventing the cells from assembling in clumps and the melanoma intravasation, extravasation and metastatic spread. Finally, we showed the inhibition of the STAT3 expression, a key protein implicated in the regulation of growth, survival, invasion, migration and metastatic spread of tumour cells [53]. Therefore, our hypothesis is that the STAT3 inhibition could also mediate the inhibition of melanoma proliferation and metastatic dissemination that we showed in treated melanoma cells.

The available chemotherapeutic drugs have a generally low effect on the growth of melanoma cells but the results that we show suggest that nanomolar concentrations of (Bu_2_Sn)_2_TPPS and (Bu_3_Sn)_4_TPPS could target multiple molecular pathways in melanoma cells. Therefore, (Bu_2_Sn)_2_TPPS and (Bu_3_Sn)_4_TPPS, for their role in the regression of melanoma motility and metastatic dissemination, could deeply interfere with the melanoma progression as new strategies for an effective treatment of this highly invasive tumour.
